# MPCaD: a multi-scale radiomics-driven framework for automated prostate cancer localization and detection

**DOI:** 10.1186/s12880-018-0258-4

**Published:** 2018-05-16

**Authors:** Farzad Khalvati, Junjie Zhang, Audrey G. Chung, Mohammad Javad Shafiee, Alexander Wong, Masoom A. Haider

**Affiliations:** 1 Department of Medical Imaging, University of Toronto and Sunnybrook Research Institute, Toronto, Ontario, Canada; 2 Department of Systems Design Engineering, University of Waterloo, Waterloo, Ontario, Canada

**Keywords:** Computer-aided detection, Multi-parametric MRI, Prostate cancer

## Abstract

**Background:**

Quantitative radiomic features provide a plethora of minable data extracted from multi-parametric magnetic resonance imaging (MP-MRI) which can be used for accurate detection and localization of prostate cancer. While most cancer detection algorithms utilize either voxel-based or region-based feature models, the complexity of prostate tumour phenotype in MP-MRI requires a more sophisticated framework to better leverage available data and exploit a priori knowledge in the field.

**Methods:**

In this paper, we present MPCaD, a novel Multi-scale radiomics-driven framework for Prostate Cancer Detection and localization which leverages radiomic feature models at different scales as well as incorporates a priori knowledge of the field. Tumour candidate localization is first performed using a statistical texture distinctiveness strategy that leverages a voxel-resolution feature model to localize tumour candidate regions. Tumour region classification via a region-resolution feature model is then performed to identify tumour regions. Both voxel-resolution and region-resolution feature models are built upon and extracted from six different MP-MRI modalities. Finally, a conditional random field framework that is driven by voxel-resolution relative ADC features is used to further refine the localization of the tumour regions in the peripheral zone to improve the accuracy of the results.

**Results:**

The proposed framework is evaluated using clinical prostate MP-MRI data from 30 patients, and results demonstrate that the proposed framework exhibits enhanced separability of cancerous and healthy tissue, as well as outperforms individual quantitative radiomics models for prostate cancer detection.

**Conclusion:**

Quantitative radiomic features extracted from MP-MRI of prostate can be utilized to detect and localize prostate cancer.

## Background

Prostate cancer is the most diagnosed cancer in Canadian men (excluding non-melanoma skin cancers), with roughly 21,300 new cases and 4100 related deaths in 2017 [1]. Nevertheless, the prognosis for prostate cancer is relatively high if it is detected sufficiently early [1]. Therefore, fast and reliable screening methods for prostate cancer are crucial.

In the current clinical method, men with a positive digital rectal exam or an elevated prostate-specific antigen (PSA) require a follow-up biopsy to assess malignancy. The PSA test in particular has recently come under scrutiny. Recent studies [2, 3] indicate that the PSA test has a high risk of overdiagnosis, with an estimated 50% of screened men being diagnosed with prostate cancer. The overdiagnosis results in expensive and painful prostate biopsies causing discomfort, possible sexual dysfunction, and increased hospital admission rates due to infectious complications [4]. The challenge diagnosticians face is how to improve prostate cancer diagnosis by reducing the overdiagnosis caused by conventional screening methods while still maintaining a high sensitivity.

Multi-parametric MRI (MP-MRI) is becoming an integral means for prostate cancer screening through which unique information is captured using different imaging modalities, thus allowing for a more comprehensive set of imaging-based features to be extracted for diagnosis. Although MP-MRI has shown considerable potential for improving prostate cancer localization accuracy [5], it requires an experienced clinician to extensively review the data and perform a diagnosis. Furthermore, there can be considerable inter-observer and intra-observer variability in imaging-based screenings [6].

Introduced recently by the European Society of Urogenital Radiology (ESUR), PI-RADS [7] (Prostate Imaging-Reporting And Diagnosis System) is a set of guidelines for interpreting multiple MR images, which aims to raise the consistency between diagnosticians through a common set of criteria. Despite PI-RADS and further development to standardize the interpretation of multi-parametric MR images [8], there is still a level of subjectiveness that can lead to inconsistent diagnosis. As such, computer-aided prostate cancer detection methods are developed to assist diagnosticians with the process and to increase not only accuracy, but reliability and consistency of diagnosis across different clinicians.

Given the need for more consistent and reliable diagnosis, a variety of computer-aided methods have been proposed for the purpose of prostate cancer detection using MP-MRI [9–14]. In particular, radiomics-driven methods for computer-aided cancer detection has emerged in recent years as holding great promise in improving diagnostic accuracy and consistency via the high-throughput extraction and utilization of a large amount of quantitative features for characterizing tumour phenotype [13–17]. While existing computer-aided cancer detection methods for prostate cancer detection using MP-MRI utilize either voxel-based or region-based feature models, the complexity of prostate tumour phenotype in MP-MRI may require a more sophisticated framework involving feature models at different scales to better leverage available data and exploit a priori knowledge in the field.

In this paper, we propose a novel Multi-scale radiomics-driven framework for automatic Prostate Cancer Detection and localization (MPCaD) that incorporates quantitative radiomics feature models characterizing tumour phenotype at different scales. Aside from proposing a unified framework for prostate cancer detection via integration of multi-scale radiomics-driven feature models, a number of novel contributions are introduced in this paper.

A voxel-resolution radiomics-driven statistical textural distinctiveness (RD-STD) method is introduced to identify tumour candidate regions. We extend significantly upon our preliminary work in [18] by incorporating a comprehensive voxel-resolution radiomics feature model composed of 96 features across five different MP-MRI modalities (i.e., T2-weighted (T2w), ADC, DWI, Computed High-B DWI (CHB-DWI), and Correlated Diffusion Imaging (CDI) [19, 20]), compared to the simple feature model composed of 19 features in [18].

A region-resolution radiomics-driven feature model (RD-FM) is introduced to facilitate for tumour region classification. This region-resolution feature model captures different regional characteristics including a multitude of morphological and textural traits to better distinguish between cancerous regions and healthy regions from the set of tumour candidate regions. We extend greatly upon the idea of a MAPS (morphology, asymmetry, physiology, and size) radiomics feature model as introduced in [21] in several significant ways.

First, the proposed RD-FM model incorporates 6 different MP-MRI modalities (i.e., T2-weighted (T2w), ADC, multiple DWI at 4 different b values, CHB-DWI, CDI, and relative ADC (a total of 9 3D image volumes per patient) compared to 4 used in the original MAPS model (i.e., T2w, ADC, DWI at a single b value, and CDI). In particular, the integration of relative ADC features are important to account for interpatient variations in ADC data. Moreover, in addition to morphology, asymmetry, and size features, the proposed RD-FM uses 26 textural features per modality compared to only 7 used in the original MAPS model (a total of 242 features per patient images compared to 42). Furthermore, the set of radiomic features comprising the proposed RD-FM model differs from that in the original MAPS model as the RD-FM model consists of optimized radiomic features chosen based on a feature selection process for a given performance criteria. A voxel-resolution relative ADC-driven conditional random field (rADC-CRF) framework is also introduced to further refine the localization of the tumour regions in the peripheral zone.

A more comprehensive set of clinical prostate MP-MRI data from 30 patients with full PI-RADS scoring and histology is introduced for assessing prostate cancer detection and localization performance, in comparison to the smaller number of patient cases used in [18] and [21] (13 patient cases and 20 patient cases, respectively).

The paper is organized as follows. The related work in the area of computer-aided prostate cancer detection is presented in “Related work” section. The methodology and underlying principles of the proposed multi-scale prostate cancer detection (MPCaD) framework are described in “Methods” section. Experimental setup and results are presented in “Results” sections and discussions are presented and future work is discussed in “Discussion” section. Finally, conclusions are drawn and discussed in “Conclusions” section.

## Related work

Current methods for automatic computer-aided prostate tumour detection typically use a supervised method trained on a set of low-level features calculated from MP-MRI. Ozer et al. [9] used parametric images derived from dynamic contrast-enhanced (DCE) MRI, and proposed the use of Relevance Vector Machines (RVM) with a Bayesian framework. Ozer et al. then evaluated the method against Support Vector Machines (SVM) with the same framework. Madabhushi et al. [22] extracted 3D texture features from MRIs where a trained Bayesian classifier assigned a malignancy “likelihood” to each feature independently, and the “likelihood” images were then combined using an optimally weighted feature combination scheme. Litjens et al. [23] proposed a two-stage prostate cancer detection algorithm via mpMRI where first, voxel-based classification was applied and a likelihood map of cancerous regions was generated. Local maxima detection was applied to the likelihood map to find local maximum with the highest probability within a predefined range (10 mm range) which yielded candidate regions via a region segmentation algorithm [23]. The candidate regions were then classified using a classifier and a feature model.

More recently, fuzzy Markov random fields (MRFs) have been investigated for prostate cancer detection as unsupervised methods [10–12]. Liu et al. [10] proposed a new method for estimating the parameters of the Markovian distribution of the measured data, and applied it to feature vectors extracted from MP-MRI prostate datasets for cancer detection. Ozer et al. [11] proposed the use of fuzzy MRFs as an unsupervised alternative to the previously proposed SVM and RVM approaches, and evaluated the classifiers using feature vectors formed from the peripheral zone of MP-MRI prostate datasets.

Artan et al. [12] presented a cost-sensitive SVM cancer localization method as an extension to the conventional SVM for prostate cancer detection. Trained via a full grid search over the SVM kernel parameters, the cost-sensitive SVM showed improvement in results compared to conventional SVM. Artan et al. [12] also proposed a new segmentation method by combining conditional random fields (CRF) with a cost-sensitive framework improving cost-sensitive SVM results by incorporating spatial information.

Recently, a particularly promising and powerful approach to computer-aided cancer detection is the concept of radiomics [13–17] with a significant potential for prostate cancer detection. Radiomics involves the high-throughput extraction and utilization of a large amount of quantitative features for characterizing tumour phenotype. Radiomics facilitates for a high-dimensional mineable feature space that can be utilized for both detection and prognosis [15]. Studies on lung and head-and-neck cancer patients have confirmed the prognostic power of radiomic features when it comes to patient outcome prediction for personalized medicine [15–17]. However, the prognostic capability of radiomics feature has only very recently been investigated for prostate cancer detection and the quantitative characterization of prostate tumour phenotype.

Khalvati et al. [14] extended the T2w- and ADC-based features from Peng et al. [24] and introduced comprehensive radiomics feature models consisting of hundreds of texture features derived from MP-MRI data via feature selection and classification for the purpose of voxel-resolution prostate tumour detection. Although [14] produced reasonable results, one limitation of such approaches is that they utilize the extracted radiomic features associated with individual voxels on an independent basis, and do not account for the overall morphological or interconnected tissue characteristics reflective of cancerous tumours. Cameron et al. [21] introduced a region-resolution feature model for prostate cancer detection where suspicious regions were initially selected by thresholding ADC and then, a feature model was applied to these candidate regions. One shortfall of this approach is its dependency on ADC threshold value to generate initial candidate regions. It has been recently shown [25] that due to interpatient variation in ADCs of prostate peripheral zone, the relative peripheral zone ADCs of the tumour and the surrounding normal regions are better correlated with cancer grade compared to peripheral zone ADC of tumour alone.

While existing computer-aided cancer detection methods for prostate cancer detection using MP-MRI utilize either voxel-based or region-based feature models, to the author’s knowledge, the incorporation of radiomics feature models at multiple scales across several MP-MRI modalities as well as the utilization of relative ADC map via a conditional random field framework for the purpose of prostate cancer detection and localization has not been previously explored and can have strong potential for improving diagnostic accuracy.

## Methods

**Fig. 1:**
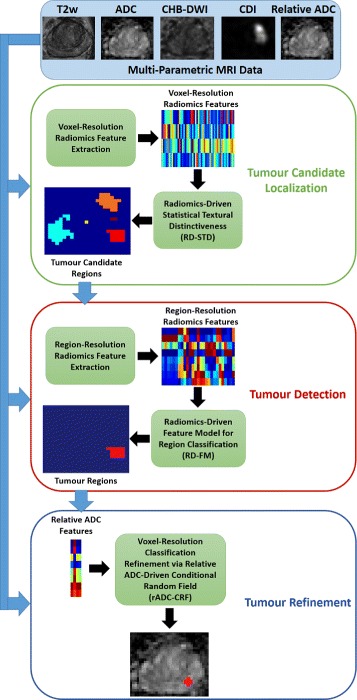
The proposed framework for automatic multi-scale prostate cancer detection (MPCaD)

Figure 1 illustrates an overview of the proposed framework for Multi-scale Prostate Cancer Detection (MPCaD). MPCaD leverages the full set of voxel-level quantitative radiomic features and incorporates region-level feature descriptors in a pipeline to better characterize and detect tumour regions.

A large amount of voxel-resolution imaging features are extracted from MP-MRI data using a quantitative radiomics feature model. Next, a classifier trained via these features is used to perform initial voxel-resolution cancer detection based on radiomics-driven statistical textural distinctiveness (RD-STD). A set of region-resolution features including morphological features are derived from the initial tumour candidate regions using a radiomics-driven feature model (RD-FM) to further distinguish between the cancerous and healthy regions. Finally, a relative ADC-driven conditional random field framework (rADC-CRF) is applied to perform voxel-resolution refinement of tumour regions produced by the previous detections, which enforces the effect of the relative ADC map on detecting cancerous regions. The detailed methodology behind each step of the proposed pipeline is described below including the imaging modalities used for both voxel-resolution and region-resolution feature models.

### Imaging methods

The main criteria for choosing imaging modalities used in the proposed framework is twofold: images that are part of PI-RADS and acquired non-invasively. PI-RADS consists of T2w, DWI (ADC) as well as DCE images. Instead of using DCE which requires contrast agent, the proposed framework uses additional information available by DWI images which includes computed high b-value image, individual b-value images, and correlated diffusion images. In addition, we compute relative ADC map to account for interpatient inconsistencies in ADC maps. These imaging methods are summarized below.

#### T2-weighted imaging (T2w)

T2w is a MR imaging modality in which the sensitivity of tissue is characterized by measuring the relaxation time (spin-spin) of the applied magnetic field. The T2w image of prostate usually shows a small reduction in signal in the cancerous tissue [5].

#### Diffusion-weighted imaging (DWI)

DWI measures the sensitivity of tissue to Brownian motion of water molecules. The signal intensity is measured by applying pairs of opposing magnetic field gradient pulses [26]. The diffusion-weighted signal, *S*, is formulated as *S*=*S*_0_*e*^−*bD*^ where *S*_0_ is the signal intensity without the diffusion weighting. The signal loss due to spins diphase is controlled by *b*, which consists of amplitude and duration of the diffusion pulses, gradient intensity and the time between the two pulses and gyromagnetic ratio, and *D* represents the strength of the diffusion.

#### Computed high-b diffusion-weighted imaging (CHB-DWI)

Although it has been shown that high b-value DWI images (e.g., b-values greater than 1000 *s*/*mm*^2^) improve the delineation between tumours and healthy tissues [27], due to hardware limitations, most MRI machines in practice do not produce DWI with b-values higher than 1500 *s*/*mm*^2^ for prostate imaging. CHB-DWI is an alternative approach to obtain high-b DWI from low b-value DWI acquisitions using a computational model [27, 28]. For our experiments, we constructed CHB-DWI from DWIs with b-values at 0,100,400,1000*s*/*mm*^2^ using a Bayesian model with the least squares estimation technique, extrapolating to the b-value of 2000*s*/*mm*^2^.

#### Correlated diffusion imaging (CDI)

CDI is a new DWI modality, which takes advantage of the joint correlation in signal attenuation across multiple gradient pulse strengths and timings to not only reduce the dependency on the way diffusion gradient pulses are applied, but also improve the separation between cancerous and healthy tissues. The CDI signal is obtained via signal mixing as follows [19, 20]:

1 $$\begin{array}{@{}rcl@{}}  CDI(x) &=& \int...\int^{b_{n}}_{b_{0}} S_{0}(x)...S_{n}(x)P(S_{0}(x),...,S_{n}(x)|\\ && V(x)) \times dS_{0}(x)...dS_{n}(x) \end{array} $$

where *x* is spatial location, *b*_*i*_ represents b values, *S* is the acquired signal, *P* is the conditional joint probability density function, and *V*(*x*) is local subvolume around *x*.

#### Relative ADC map

Discovered recently, due to interpatient variation in ADC of prostate peripheral zone, relative peripheral zone ADCs of tumour and surrounding normal regions are better correlated with cancer grade compared to peripheral zone ADC of tumour alone [25]. To compensate for interpatient variation in ADC, the relationship between ADC values of tumour regions and surrounding regions can be used to refine the result of tumour candidate classification. First, we create a *relative ADC map*, which takes this phenomenon into account. Next, we apply conditional random field to enforce the effect of relative ADC on tumour candidate classification.

For a given tumour candidate region of interest (ROI), we first used morphological dilation function (with structuring element of *SE*) to enlarge the ROI and then subtract it from the original ROI. This gives median value for the surrounding region of the original ROI. Next, the dilated (enlarged) ROI was normalized by the median value of the surrounding region. Once this was done for each candidate ROI, all normalized dilated ROIs were combined together and then replaced the original dilated ROIs in the ADC map. The final map was normalized once more with respect to the mean value of median surrounding regions of all candidate ROIs. Algorithm 1 lists the steps for generating relative ADC map.



### Tumour candidate region identification via Voxel-resolution Radiomics-driven statistical textural distinctiveness (RD-STD)

Similar to [18], we identified suspicious prostate tissue via statistical textural distinctiveness using cross-modality texture features extracted from MP-MRI data. Voxel-resolution texture representations were used to capture healthy and cancerous prostate tissue characteristics present in MR imaging. The texture feature model proposed in [14] was used to extract 96 low-level texture features derived from T2w images, ADC maps, CHB-DW data, and CDI data. Table 1 summarizes all features used in this feature model.

**Table 1:** Summary of feature groups in proposed Radiomics-Driven Statistical Textural Distinctiveness (RD-STD) [14]

Feature group	Number of features	Description
Textural (1^*st*^-order)	4	Mean, Standard deviation, Kurtosis, Skewness
		Energy, contrast, correlation, variance, inverse difference moment normalized,
Textural (2^*nd*^-order)	72	Sum average, sum variance, entropy, sum entropy, difference entropy,
	(18 in each of 4 directions)	Information measure of correlation, homogeneity, autocorrelation
		Difference variance, dissimilarity, cluster shade, cluster prominence, maximum probability
Gabor filters	12	3 scales and 4 orientations
Kirsch filters	8	8 directions
Total	96	All features

Given the full set of texture features *h*(*x*), a compact version *t*(*x*) is generated using the *u* number of principal components of *h*(*x*) via principal component analysis (PCA):

2 $$ t(x) = \langle \Phi_{i}(h(x)) | 1 \leq i \leq u \rangle   $$

where *Φ*_*i*_ is the *i*^th^ principal component of *h*(*x*). As [18] determined through empirical testing, *u* components of *h*(*x*) were selected to represent 90% of the variance of all the textural representations.

Given textural representations of the prostate tissue, a sparse texture model is learned to characterize healthy and suspicious tissues. A global texture model is defined using *t*(*x*) representing the characteristics of healthy and cancerous prostate tissues. However, an MRI slice can be generalized as a set of regions where a unique texture pattern is repeated in each one. Furthermore, global texture modeling is computationally expensive which leads us to incorporate a sparse texture modeling framework where the number of regions of unique textures is significantly fewer than the number of voxels in *t*(*x*). As such, a sparse texture model comprising of a small set of *m* local texture representations can be defined as follows:

3 $$ T^{r} = \left\{ t_{i}^{r} | 1 \leq i \leq m \right\}   $$

where each texture atom represents the mean and covariance (i.e., $t^{r}_{i} = {\underline {\mu }_{i},\Sigma _{i}}$) of a local texture representation, and is learned via expectation maximization [29].

Suspicious regions in prostate tissue are unique and texturally distinct relative to healthy tissue. The distinctiveness of these texture patterns can be quantified using the concept of statistical textural distinctiveness [30]. The Kullback—Leibler (KL) divergence [31] is used to define the statistical textural distinctiveness between two local texture representations (denoted as $t_{i}^{r}$ and $t_{j}^{r}$) in the sparse texture model:

4 $$\begin{array}{@{}rcl@{}} \beta_{i,j} &=& \log\frac{|\Sigma_{j}|}{|\Sigma_{i}|} - u + trace\left(\Sigma_{j}^{-1}\Sigma_{i}\right)\\  && + \frac{\left(\underline{\mu}_{j} - \underline{\mu}_{i}\right)^{T}\Sigma_{j}^{-1}\left(\underline{\mu}_{j} - \underline{\mu}_{i}\right)}{2} \end{array} $$

where *u* is the number of PCA components selected, $\underline {\mu }_{i}$ and $\underline {\mu }_{j}$ represent the mean of $t_{i}^{r}$ and $t_{j}^{r}$, respectively, and *Σ*_*i*_ and *Σ*_*j*_ represent the covariance of $t_{i}^{r}$ and $t_{j}^{r}$, respectively. As such, the distinctiveness metric *β*_*i*,*j*_ increases as the texture patterns of $t_{i}^{r}$ and $t_{j}^{r}$ become more distinct from one another.

Due to the uniqueness and statistical occurrence of the corresponding texture characteristics, salient regions in prostate MRI data can be interpreted as suspicious as the majority of prostate tissue is typically healthy. Given a test set of texture features *t*(*x*)_*Z*_ extracted from MRI slices, the saliency map for an MRI slice can be computed using the learned sparse texture model where for $P\left (t^{r}_{i} | Z\right)$ (the occurrence probability of $t_{i}^{r}$ in *t*(*x*)_*Z*_), saliency *α*_*i*_ is computed as:

5 $$ \alpha_{i} = \sum^{m}_{j = 1} \beta_{i,j} P\left(t^{r}_{i} | t(x)_{Z}\right).   $$

For saliency *α*_*i*_, the voxels in the corresponding set of texture representations *S*_*i*_ are considered salient (and therefore classified as suspicious tissue) given that $\alpha _{i} > \frac {\alpha _{max}}{2}$, and all other voxels are classified as healthy. Therefore, each voxel *x* in a test set is assigned a label *y*:

6 $$\begin{array}{*{20}l} y = \left\{\begin{array}{ll} 1 & x \in S_{i}, \alpha_{i} > \frac{\alpha_{max}}{2} \\ 0 & otherwise \end{array}\right. \end{array} $$

### Tumour region selection via a region-resolution Radiomics-driven feature model (RD-FM)

Once tumour candidate regions were identified, the next step in the proposed MPCaD framework is to develop a quantitative radiomics feature model that extracts features from each candidate regions to determine which regions are more likely cancerous. The radiomics feature model proposed here is based on a greatly extended version of MAPS model introduced in [21]. The MAPS model calculates different types of features representing Morphology, Asymmetry, Physiology, and Size of each candidate region. While the proposed RD-FM model incorporates 6 different MP-MRI modalities (i.e., T2w, ADC, multiple DWI at 4 different b values, CHB-DWI, CDI, and relative ADC), the original MAPS model used only 4 (i.e., T2w, ADC, DWI at a single b value, and CDI). In addition, the proposed RD-FM uses 26 textural features per modality compared to only 7 used in the original MAPS model.

In contrast to [21] where the physiology features were calculated for a local sliding window within the candidate region and then averaged across all windows, here, we calculate these features with respect to the entire candidate region as a global feature. The motivation behind this approach is the fact that textural characteristics of tissue are better represented when the entire region is taken into account rather than a local window (e.g., 3×3). For example, while patches of a candidate region may appear homogeneous, when the entire candidate region is considered, it may be a heterogeneous region, which is an important characteristic for cancerous regions.

Once the features are calculated for candidate regions, in contrast to the original MAPS, a feature selection method is applied to select best features in terms of the ability to separate the cancerous regions from non-cancerous ones. The selected best features are then fed into a SVM classifier. We adopted the method proposed in [14] to optimize the results of the feature model (RD-FM) for specificity, sensitivity, and AUC using different feature selection criteria.

#### MAPS feature model

MAPS feature model consists of 4 feature categories: morphology, asymmetry, physiology, and size. While ***m***orphology, ***a***symmetry, and ***s***ize (*F*_*mas*_) depend on the shape and size of the tumour candidate regions, ***p***hysiology features (*F*_*p*_) extract textural characteristics of the regions. Therefore, morphology, asymmetry, and size features are independent of the imaging modality under study whereas the physiology features are extracted from different imaging modalities as listed below:

- *I*_1_ = T2w
- *I*_2_ = ADC
- *I*_3_ = Relative ADC
- *I*_4_ = CHB-DWI: b-value at 2000*s*/*mm*^2^
- *I*_5_ = CDI
- *I*_6_=*b*_1_: b-value at 0*s*/*mm*^2^
- *I*_7_=*b*_2_: b-value at 100*s*/*mm*^2^
- *I*_8_=*b*_3_: b-value at 400*s*/*mm*^2^
- *I*_9_=*b*_4_: b-value at 1000*s*/*mm*^2^

##### Morphology

A set of features are computed to characterize the structural property and morphology of tumour candidate regions. Three morphological features are computed from the region boundary. We calculate the first morphology feature as the normalized difference in area between the morphological closing and opening of the region, using an identical disk structuring element [32]:

7 $$  f_{1}^{M} = \frac{A_{closed} - A_{opened}}{A_{initial}}  $$

where *A* denotes the area of a region. Peaks and valleys in the border of the region will cause the area to increase after closing, while it will decrease after opening. Thus, regions with irregular borders will have a greater difference between these two values, and the feature value will be larger.

The second morphological feature is calculated as the normalized difference between the length of the region’s perimeter before and after eliminating high-frequency components in the Fourier space:

8 $$  f_{2}^{M} = \frac{|P_{initial} - P_{reconstruction}|}{P_{initial}} \,,  $$

where *P* is the perimeter of each region. Since high-frequency components capture rapid changes in the region shape, this feature will be greater for regions with rapidly-varying boundaries than for those with smooth, slowly-varying boundaries [32].

The third morphological feature examines the area difference between two Fourier reconstructions of the region boundary, one at a low frequency and one at a higher frequency:

9 $$  f_{3}^{M} = \frac{\left\vert{A_{initial} \oplus A_{reconstruction}}\right\vert} {\left\vert{A_{initial} \cup A_{reconstruction}}\right\vert}  $$

where ⊕ represents the set symmetric difference, finding voxels which are in the low-frequency reconstruction or the high-frequency one, but not in both, normalized by the area of the union of both reconstructions, denoted by ∪.

##### Asymmetry

The Asymmetry feature group represents the degree of bilateral symmetry of a candidate region, which is calculated by splitting the region in half along an axis passing through its center of mass. The halves to either side of this axis are then compared by taking the difference in their areas, and normalizing it:

10 $$  f^{A}_{\circ} = \frac{A_{large} - A_{small}}{A_{normalize}}  $$

where *A*_*large*_ and *A*_*small*_ represent the areas of the region halves, chosen such that *A*_*large*_≥*A*_*small*_, and *A*_*normalize*_ represents the area of the region used to normalize the difference. Four different Asymmetry features are computed by choosing either the major or the minor axis to split the region, and by choosing either the entire region area or the area of the smaller half-region as *A*_*normalize*_.

##### Physiology

The physiology features include the first order and second order statistical features. For first order features, the pixels within the ROI are used to calculate the features. To calculate second-order features, gray-level co-occurrence matrix (GLCM) is calculated in horizontal direction for adjacent pixels. To account for only those pixels within the ROI, when calculating the GLCM, it is verified whether both adjacent pixels belong to the ROI. Table 2 summarizes all features used in the MAPS feature model.

**Table 2:** Summary of feature groups in proposed Radiomics-Driven Feature Model (RD-FM) [21]

Feature group	Number of features	Description
Morphology	3	Area regularity (1), Perimeter regularity (2)
Asymmetry	4	Region bilateral symmetry (4)
Physiology	26	
Textural (1^*st*^-order)	7	Mean, median, standard deviation, minimum, maximum, kurtosis, skewness
		Energy, contrast, correlation, variance, inverse difference moment normalized, sum average,
Textural (2^*nd*^-order)	19	Sum variance, entropy, sum entropy, difference entropy, normalized entropy,
		Information measure of correlation, homogeneity, difference variance,
		Autocorrelation, dissimilarity, cluster shade, cluster prominence, maximum probability
Size	1	Size of region
Total	34	All features

In total, for each ROI, 8 features are extracted for morphology, asymmetry, and size features (*F*_*mas*_) and 26 features are extracted for physiology features (*F*_*p*_). As discussed, *F*_*mas*_ features are independent of imaging modality and depend on the shape and size of the ROI. In contrast, for a given ROI, the *F*_*p*_ features will be different for each imaging modality. Therefore, each ROI will have 242 MAPS features in total (morphology 3, asymmetry 4, size 1, physiology 234=26×9 where 9 is the number of imaging modalities).

#### Feature selection model

We apply a feature selection method to choose the features that contribute the most to the classification process. For feature extraction function, we used the maximum relevance, minimum redundancy (mRMR) technique [33], which is based on maximum relevance and minimum redundancy of features. We evaluate the selected features with respect to sensitivity, specificity, and the area under the ROC (Receiver operating characteristic) curve. In other words, for a given feature group, the optimal feature subset is the ones that maximize the desired evaluation measure (e.g., area under the ROC curve).

**Fig. 2:**
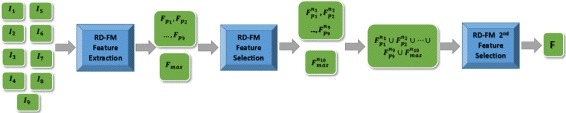
Block diagram of the proposed radiomics-driven feature model (RD-FM).

Each imaging modalities (*I*_*i*_) is used to generate physiology features ($F_{p_{i}}$) and the feature selection method is then applied to each feature set of each imaging modality $F_{p_{i}}$ to determine the optimal subset features for that modality ($F_{p_{i}}^{n_{i}}$). For imaging modality *I*_*i*_, $F_{p_{i}}$ is the original physiology feature set and $F_{p_{i}}^{n_{i}}$ is *n*_*i*_ optimal physiology features which maximize the desired evaluation measure. For morphology, asymmetry, and size features, they are grouped together as *F*_*mas*_ and then feature selection is applied to pick the best reduced feature set. This produces *n* features ($F_{mas}^{n}$) which maximizes the desired evaluation measure. These optimal subset of features ($F_{p_{1}}^{n_{1}}$, $F_{p_{2}}^{n_{2}}$,..., $F_{p_{9}}^{n_{9}}$, and $F_{mas}^{n}$) are combined together and the feature selection method is applied again to find the final subset of features (*F*) that maximize the desired evaluation measure when combined together. Figure 2 shows the block diagram of the proposed feature selection for RD-FM.

### Tumour region modification using relative ADC-driven conditional random field (rADC-CRF)

Once the tumour candidate regions were selected by RD-FM, we apply a relative ADC-driven conditional random field (rADC-CRF) method to perform final voxel-resolution cancer detection refinement enforcing the relative ADC on the detection results. Conditional random fields were first proposed by Lafferty et al. [34] and have previously been used for image labelling [35]. In addition, in [36], it was shown that CRF can be used to enforce the spatial constraints on prostate tumours such as compactness.

Here, we extend upon the CRF model proposed in [36] to leverage the full set of voxel-level quantitative radiomic features derived from relative ADC data while taking into account the spatial relationships and quantitative radiomics feature relationships between voxels to better enforce relative ADC characteristics. The CRF framework models the conditional probability of the binary label field *Y* and corresponding *X* observations as follows [36]:

11 $$\begin{array}{*{20}l} P(Y | X) = \frac{1}{Z(X)}\exp(-E(Y,X))  \end{array} $$

where *Z* and *E* represent the normalizing and energy functions, respectively. The prostate tissue labeling as healthy or cancerous is optimized using a Maximum A Posteriori (MAP) method where the best classification of healthy and tumour tissues are achieved by minimizing the energy function *E*:

12 $$\begin{array}{*{20}l} Y^{*} = \underset{Y}{\arg\min} E(Y,X)  \end{array} $$

where *Y*^∗^ is the optimal solution given the patient’s relative ADC map. *E*(*Y*,*X*) is formulated as a combination of unary and pairwise potential functions:

13 $$\begin{array}{*{20}l} E(Y,X) = \sum^{n}_{i=1} \psi_{u}(y_{i},X) + \sum_{\varphi \in C} \psi_{p}(y_{\varphi},X)  \end{array} $$

*E* incorporates the data-driven unary function *ψ*_*u*_ which is the results of RD-FM classification and pairwise function *ψ*_*p*_, which contains inter-voxel radiomic features extracted from relative ADC map across a set of clique structures *C*. To obtain the final voxel-resolution tumour detection results, the energy function *E* is minimized using gradient descent, and the binary label is assigned to each voxel as *y*^∗^∈*Y*^∗^.

By taking full advantage of 96 voxel-resolution radiomic features (similar to RD-STD) extracted from relative ADC map, the final voxel-resolution cancer detection is improved.

## Results

In this section, the image data, quantitative results for MPCaD, and the comparison with related work are presented.

### Image data

The performance of the proposed MPCaD was evaluated using clinical MP-MRI data of 30 patients (17 with cancer and 13 without cancer) acquired using a Philips Achieva 3.0T machine at Sunnybrook Health Sciences Centre (SHSC), Toronto, ON, Canada. The mean patients’ age was 62±9 years. All data was obtained retrospectively under the local institutional research ethics board. For each patient, the following MP-MRI modalities was obtained (Table 3): 1) T2w, ii) DWI, and iii) CDI. Images were processed in the ProCanVAS (Prostate Cancer Visualization and Analysis System) platform developed at SHSC [37].

**Table 3:** Description of the prostate imaging data

Modality	DFOV (*cm*^2^)	Resolution (*mm*^3^)	TE (ms)	TR (ms)
T2w	22×22	0.49×0.49×3	110	4687
DWI	20×20	1.56×1.56×3	61	6178
CDI	20×20	1.56×1.56×3	61	6178

All imaging data were reviewed and marked as healthy and cancerous by a radiologist with 20 and 15 years of experience interpreting body and prostate MRI, respectively. Using the biopsy location reported in the pathology reports, the experienced radiologist compared the MP-MRI images with the histopathology images/reports marked by a pathologist as cancer (i.e., Gleason score 6 and above) and annotated the cancerous regions on MP-MRI data. These regions corresponded to PI-RADS scores of 3 and above. For these 17 cases with cancer, 4 cases had 2 tumours and the remaining 13 had one tumour (21 tumours in total). For these 21 tumourous regions, the PI-RADS scores were as follows: PI-RADS score 5 (14 cases), PI-RADS score 4 (4 cases), and PI-RADS score 3 (3 cases). For the 13 non-cancerous cases, the PI-RADS scores were 2.

### Experimental setup

To assess the efficacy of the proposed framework and evaluate the effect of each pipeline stage on the prostate cancer detection performance, we conducted a set of analysis using the methods detailed in “Methods” section. First, radiomics-driven statistical textural distinctiveness (RD-STD) was calculated to separate suspicious cancerous voxels from healthy voxels using leave-one-patient-out cross validation where on average, 212,000 and 7300 voxel-resolution feature vectors were used for training and testing, respectively. Then, a radiomics-driven region-resolution quantitative feature model (RD-FM) was applied to the candidate regions detected in the first step to further distinguish cancerous from healthy regions using leave-one-patient-out cross validation where on average, 1183 and 41 region-resolution feature vectors were used for training and testing, respectively. In this step, we also applied different feature selection criteria to the radiomics feature model to assess their effects on the detection results. Finally, a relative ADC-driven conditional random field framework (rADC-CRF) was utilized to refine the produced tumour regions again at voxel-level enforcing the relative ADC values.

### Quantitative results

Table 4 shows the detection results of the three sequential procedures in the proposed MPCaD framework. RD-STD produced the highest sensitivity of 0.92, coupled with a low specificity of 0.07 and a low accuracy of 0.17. In the second step, the best performance of RD-FM considerably improved the specificity from 0.07 to 0.89, and achieved a sensitivity and accuracy of 0.85. rADC-CRF refined the results produced by RD-FM and further increased the specificity to 0.90, and the accuracy to 0.86, while maintaining sensitively at 0.85.

**Table 4:** Evaluation results of each stage in MPCaD (Results are shown with 95% confidence interval)

Procedure	Feature Selection Criteria	Sensitivity	Specificity	Accuracy
RD-STD	-	**0.92** [0.84 0.99]	0.07 [-0.02 0.16]	0.17 [0.08 0.27]
RD-FM	Specificity	0.79 [0.67 0.91]	**0.89** [0.85 0.93]	**0.85** [0.80 0.90]
	Sensitivity	**0.85** [0.74 0.96]	0.86 [0.81 0.91]	0.84 [0.78 0.90]
	AUC	0.83 [0.71 0.95]	0.83 [0.76 0.90]	0.83 [0.78 0.89]
	**Average**	0.82	0.86	0.84
rADC-CRF	Specificity	0.79 [0.63 0.95]	**0.90** [0.86 0.94]	**0.86** [0.82 0.90]
	Sensitivity	**0.85** [0.70 1.00]	0.87 [0.82 0.92]	0.85 [0.80 0.90]
	AUC	0.83 [0.66 0.99]	0.88 [0.83 0.93]	0.85 [0.81 0.89]
	**Average**	0.82	**0.89**	**0.86**

The bold font shows the best result

The results indicate a high percentage of tumour candidate regions are detected in the first RD-STD step (602), but a large number of false positives exist in this voxel-resolution detection (536 or 89%). These false positives are effectively reduced by the following RD-FM radiomics feature model (79 or 13%), which utilizes region-resolution radiomics and enables to further distinguish between cancerous and healthy tissue in the candidate regions. The detection is finally refined at voxel-level by the rADC-CRF framework (72 or 12% false positives), incorporating spatial and radiomics feature relationship between relative ADC voxels.

The second and third blocks in Table 4 give details on the detection results of RD-FM and rADC-CRF, respectively, using different feature selection criteria. Adopted from [14], the RD-FM enables the selection of the best subsets of features to perform the classification to maximize the corresponding performance evaluation metrics. In our experiments, we examined three feature selection criteria namely to maximize specificity, sensitivity and area under curve (AUC) for ROC curve. As it can be seen in Table 4, when the feature selection criteria is to maximize specificity or sensitivity, RD-FM followed by rADC-CRF generates the highest specificity (0.90) or sensitivity (0.85). Using specificity as feature selection criteria also gives the highest accuracy (0.86) compared to those using sensitivity (0.85) and AUC (0.85). When using AUC as feature selection criteria, RD-FM followed by rADC-CRF produced a balanced result in terms of sensitivity (0.83) and specificity (0.88).

rADC-CRF improves the specificity while maintaining the sensitivity. Choosing specificity as the feature selection criteria, rADC-CRF stage increased the specificity from 0.89 to 0.90 and accuracy from 0.85 to 0.86 with the sensitivity remaining at 0.79. Choosing sensitivity as the features selection criteria, rADC-CRF stage increased the specificity from 0.86 to 0.87 and accuracy from 0.84 to 0.85 while maintaining sensitivity at 0.85. Finally, choosing area under ROC curve as the feature selection criteria, rADC-CRF stage increased the specificity from 0.83 to 0.88 and accuracy from 0.83 to 0.85 with sensitivity remaining at 0.83. If the results for all 3 feature selection criteria are averaged, it is seen that rADC-CRF stage increased the specificity from 0.86 to 0.89 and accuracy from 0.84 to 0.86 while maintaining sensitivity at 0.82.

**Fig. 3:**
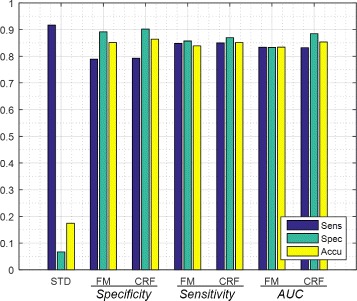
MPCaD framework results. Legend denotes the result metrics and the grouping in x axis (e.g., AUC) shows the feature selection criteria used for experiments

Figure 3 gives an overview of the performances of different stages in the framework, with RD-FM and rADC-CRF grouped by the feature selection criteria used in RD-FM, as rADC-CRF was built on RD-FM results. There is a dramatic improvement on specificity from RD-STD to RD-FM as shown in the graph, which indicates radiomic features at region-resolution has great separability for cancerous and healthy tissue. It is interesting to observe how the performance metrics evolves from RD-FM to rADC-CRF, which exhibits the same pattern in all three groups. Specificities and accuracies of rADC-CRF improve over RD-FM across all cases by, on average, about 3% and 2%, while sensitivities remains unchanged.

To summarize, RD-STD produced the highest sensitivity (0.92), aligning with our purpose to detect as many tumour candidate regions as possible in the first stage. RD-FM using specificity as feature select criteria (FM-SPEC) maximized the detection for specificity (0.89) and produced the highest accuracy (0.85) with sensitivity of 0.79. RD-FM with sensitivity as feature select criteria (FM-SENS) resulted in the highest sensitivity (0.85) with reasonable specificity (0.86) and accuracy (0.84). rADC-CRF based on FM-SPEC further refined the results and produced the highest specificity (0.90) and accuracy (0.86) with maintaining sensitivity (0.79).

The results given here are based on regions detected by the RD-STD stage, and classified by the RD-FM and rADC-CRF stages. In practical scenarios, the end-goal is usually to determine whether or not a patient has cancer, independent of regions within 3D volume of MP-MRI data. To achieve this, we only look at the regions that MPCaD determined to be positive for each patient (i.e., true positive and false positive regions combined). Using a simple threshold (i.e., how many regions should be positive to call a patient cancerous), we were able to achieve sensitivity, specificity, and accuracy of 0.76, 0.92, 0.83, respectively at patient level. In other words, out of 30 patients (17 with cancer and 13 without cancer), we were able to correctly detect 13 patients out of 17 as cancerous, with only one false positive (healthy patient detected as cancerous).

**Fig. 4:**
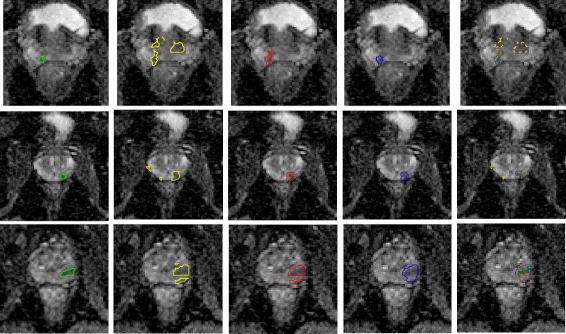
Visual comparison of identified prostate tumour candidates in each stage of the pipeline for 3 cases. Left to right: radiologist’s markings (green), results produced by RD-STD (yellow), RD-FM (red), rADC-CRF (blue), and all results shown in one image

Figure 4 shows the prostate tumour candidates detected in each stage of MPCaD compared to the ground-truth regions. It clearly presents the detection improvements through the different stages of the pipeline by utilizing radiomic features at different resolutions. Second column shows that via voxel-level detection, RD-STD captured more suspicious tumour regions (e.g., 4) than the ground truth (1). As shown in the third column, RD-FM correctly removed the 3 false positives and left only the true positive, by using region-based radiomics. Shown in the forth column, rADC-CRF made further refinement on voxel-level of the result. The final result shows that the cancerous region aligns better with the tumour contours of the radiologist’s marking.

### Comparison with other methods

MPCaD performance was compared to the original MAPS model [21] as well as the most commonly used MP-MRI that is noninvasive (T2w+ADC [24]). For T2w+ADC, we modified the original algorithm of [24] where it was fed with the tumour candidate regions detected by RD-STD model followed by a texture feature model of T2w and ADC images. The proposed MPCaD framework outperformed both the original MAPS model [21] and the conventional MP-MRI of T2w+ADC model (i.e., Peng et al. [24]) in all metrics (Table 5).

**Table 5:** Performance comparison with previous work

Method	Sensitivity	Specificity	Accuracy
MPCaD	**0.82**	**0.89**	**0.86**
MAPS [21]	0.51	0.81	0.70
Peng et al. [24]	0.78	0.75	0.74

The bold font shows the best result

## Discussion

Medical imaging is increasingly used in clinical practice for diagnosis and treatment guidance of prostate cancer, for its ability to assess the characteristics of human tissue noninvasively. Automated prostate cancer detection using MP-MRI is investigated actively and shows great promise for the future in the diagnosis, treatment, and monitoring of prostate cancer. Compared to conventional MRI, MP-MRI enables better anatomical delineation, improved specificity in characterization of lesion, and a more reliable assessment of organ confinement of the tumour to guide therapy [38]. As the first major contribution, in addition to T2w and DWI, in this work, we have also incorporated information from all available MR image data including different b-value images of DWI (i.e. b-values at 0, 100, 400, and 1000 *s*/*mm*^2^). We have also incorporated two extra image modalities namely CHB-DWI [27] and CDI [19] to enrich our model in terms of data diversity. Furthermore, we have used relative ADC map to account for interpatient inconsistencies in ADCs when it comes to separability of cancerous and healthy tissues. Thus, the proposed framework utilizes a comprehensive set of MP-MRI modalities for accurate detection and localization of prostate cancer.

Radiomics attempts to quantify tumour phenotypes by applying a large number of quantitative image features either voxel-based [14] or region-based [21]. As the second major contribution, this paper presents a framework for prostate cancer detection by integrating multiple feature models and taking full advantage of radiomic features extracted at different resolutions/scales. The proposed framework first implements an initial detection of cancerous and healthy tissues based on statistical distinctiveness at voxel-resolution (RD-STD). Then, it adopts a region-resolution radiomics feature model to further distinguish tumour and healthy tissues in the candidate regions (RD-FM). Region-based approach is more robust to image noise and can produce less sparsely distributed tumour candidates than voxel-based approach. The multi-scale nature of the proposed framework improves the separability of cancerous and healthy tissue through the pipeline and outperforms individual single-scale radiomics methods (i.e., voxel-based on region-based alone).

As another major contribution, the RD-FM presented in this paper differs from the initial work [21] in several aspects. The first is how tumour candidate regions are prepared. In [21], an empirical threshold (e.g., 700) was applied to ADC map to filter out tumour candidate regions, which is both subjective and cannot be widely used due to variability in image acquisition across scanners and interpatient inconsistency. In contrast, in this paper, we detect tumour candidate regions from healthy tissue through a statistical textural distinctiveness method, in which the feature distributions of tumour and healthy tissue are learned from the image data directly. In this respect, this method is more objective and holds greater translational potential. Another aspect lies in how the region-based features are extracted. In [21], physiology features were calculated for a local sliding window within ROI and then average across all windows. In our approach, we calculate these features with respect to the entire ROI as a global feature, which better represents the textural characteristics of the regions. We also integrate new features extracted from relative ADC map to account for interpatient variation in ADC maps. In addition, the proposed RD-FM uses 26 textural features compared to only 7 used in the original MAPS model. Finally, similar to the RD-STD stage, in addition to 4 imaging modalities used in [21] (T2w, ADC, CHB-DWI, and CDI), RD-FM also use 4 individual b-value images of DWI and relative ADC to extract features.

With a feature selection radiomics model in its core, another distinctive aspect of the proposed framework is that it can be easily configured to optimize for sensitivity, specificity, or the area under the ROC curve, to fit the targeted clinical procedure which imposes different performance requirements. For example, cancer screening programs require high sensitivity. In such cases, we can configure the model to use sensitivity as the performance evaluation criteria to steer the feature selection process which would lead to the best result for sensitivity (e.g., 0.85) with reasonable results for specificity (e.g., 0.87). For cases where higher specificity is required (i.e., radical prostatectomy), one can use specificity as the performance evaluation criteria to optimize the results for specificity (e.g., 0.90) with acceptable sensitivity (e.g., 0.79).

Finally, the last major contribution in the proposed MPCad is the utilization of a conditional random field (CRF) framework to incorporate the interpatient variation in ADC maps as well as the enforcement of connectedness of cancerous regions in prostate. This is done by further refining the results from RD-FM stage at voxel-resolution through a CRF framework (rADC-CRF) to reinforce the relative ADC map effect on the tumour region detection. Previously, it was reported [36] that a CRF framework can noticeably reduce the sparsely distributed tumour candidates on results produced by voxel-based approach. In our experiment, the CRF model is applied to relative ADC map and results show it further increases the specificity by reducing the number of false positive. It is important to note that the CRF model incorporates not only the spatial relationships between detection results from RD-FM at voxel-resolution, but the quantitative radiomics feature relationships between voxels in the relative ADC map as well. This facilitates for the enforcement of interconnected tissue characteristics reflective of cancerous tumours, thus better representing the actual cancerous tissue phenotype.

The comparison of the proposed MPCaD with the original MAPS model [21] and a conventional MP-MRI model which included T2w and ADC textural features [24] showed the superior performance of the proposed MPCaD framework.

Given that in the clinical workflow, it is important to determine whether or not a patient has cancer, our results show that the proposed MPCaD framework can accurately achieve this: out of 17 patients with cancer, 13 were detected correctly with only 1 false positive case. This confirms the capability of the proposed framework to be used in real-world scenarios.

Future work for the proposed framework includes improving the tumour candidate region identification method. Sensitivity of the pipeline, to a large extend, is limited by the tumour candidate region identification method in the first step. If a tumour region is undetected in the first step, it is hard to gain it back in the following steps, if not impossible. We will investigate other tumour candidate region generation methods to improve the sensitivity, and detect as many tumour candidate regions as possible in the first step. Superpixel segmentation is a good candidate and shows great promise in generating intra-segment homogenous, regular shaped and sized regions, which are ideal in serving as structural elements of both cancerous and healthy regions [39]. Furthermore, future work also includes evaluating the proposed framework on a larger and more diversified dataset to investigate the proposed pipeline more thoroughly. In addition, the robustness of the proposed framework to inter-observer and intra-observer variability in annotation of the ground-truth data will be evaluated using the markings of several radiologists on MP-MRI as well as markings of the same radiologist performed twice on the same dataset with a sufficient time interval (e.g., 3 weeks).

In recent years, deep neural networks have demonstrated effectiveness in performing various vision tasks including medical image analysis [40]. For instance, a method proposed by Zhu et al. [41] showed that latent high-level features learned by a Stacked Auto-Encoder model can be added to the conventional handcrafted features to achieve better prostate cancer detection performance. As a next step to the proposed method in this paper, we will incorporate deep convolutional neural networks (CNNs) into a computer-aided prostate cancer detection framework where CNNs-driven features are combined with conventional radiomic features for improved detection of prostate cancer. This would enable the utilization of both hand-crafted features, which have been designed to capture specific characteristics of images, and intrinsic features extracted automatically based on the property of a given training dataset leading to improved performance.

## Conclusions

In this paper, we introduced a novel framework for automatic detection of prostate cancer via a multi-resolution radiomics feature model pipeline which extracts and exploits comprehensive sets of features from multiple modalities in MP-MRI. The proposed framework leverages the full set of voxel-level quantitative radiomic features and incorporates region-level features to better characterize and detect tumour regions. A radiomics-driven conditional random field is introduced in the last stage of the pipeline to enforce relative ADC values by incorporating spatial and radiomics feature relationships between voxels to improve detection results. The conditional random field framework also enforces the connectednes of cancerous regions in prostate and hence, better representing the actual cancerous tissue phenotype. A unique advantage of the proposed framework is the flexibility to optimize for different performance metrics of specificity, sensitivity, or area under the ROC curve to fit the targeted clinical procedure. The proposed framework shows promise as a computer-aided diagnosis tool for accurate and consistent diagnosis of prostate cancer with a great potential for image-guided treatment procedures.

## Abbreviations

MPCaD: Multi-scale radiomics-driven framework for Prostate Cancer Detection MP-MRI: Multi-parametric magnetic resonance imaging ADC: Apparent diffusion coefficient PSA: prostate-specific antigen ESUR: European Society of Urogenital Radiology PI-RADS: Prostate Imaging-Reporting And Diagnosis System RD-STD: Radiomics-driven statistical textural distinctiveness DWI: Diffusion-weighted imaging CHB-DWI: Computed high-b diffusion-weighted imaging CDI: Correlated diffusion imaging (CDI) RD-FM: Region-resolution radiomics-driven feature model MAPS: Morphology, asymmetry, physiology, and size rADC-CRF: Relative ADC-driven conditional random field CRF: Conditional random field DCE: Dynamic contrast enhanced T2w: T2-weighted imaging ROI: Region of interest SE: Structuring element PCA: Principal component analysis ROC: Receiver operating characteristic AUC: Area under curve CNNs: Convolutional neural networks
